# Amyloidosis in Human Inborn Errors of Immunity Predicts Poor Prognosis

**DOI:** 10.1007/s10875-025-01875-1

**Published:** 2025-04-23

**Authors:** Elif Soyak Aytekin, Anar Tagiyev, Onat Silleli, İncinur Samur, Fevzi Demirel, Saliha Esenboğa, Emine Arzu Sağlam, Deniz Çağdaş

**Affiliations:** 1 Department of Pediatric Immunology, Hacettepe University Medical School, Ankara, Türkiye Turkey; 2 E. Qarayev Children Hospital, Baku, Azerbaijan; 3 Hacettepe University Medical School, Ankara, Türkiye Turkey; 4 Division of Immunology and Allergic Diseases, Gülhane Training and Research Hospital, Ankara, Türkiye Turkey; 5 Department of Pathology, Hacettepe University Medical School, Ankara, Türkiye Turkey; 6 Department of Pediatrics Section of Pediatric Immunology, Hacettepe University Medical School İhsan Doğramacı Children’s Hospital Institute of Child Health, Altındağ, Ankara, 06100 Turkey

**Keywords:** AA amyloidosis, Colchicine, Immune dysregulation, Inborn errors of immunity, Renal involvement

## Abstract

**Purpose:**

Chronic inflammation in inborn errors of immunity(IEI) caused by the infections or immune dysregulation is associated with the amyloid A (AA) amyloidosis development. This study aims to analyze the clinical characteristics, management strategies, and outcomes of patients with IEI complicated by AA amyloidosis, focusing on demographics, disease manifestations, treatment modalities, and survival rates.

**Methods:**

Thirteen patients diagnosed with IEI and AA amyloidosis, along with an additional 10 patients previously reported from Türkiye, were reviewed retrospectively.

**Results:**

The median ages at diagnosis of IEI and amyloidosis were 20 years (2–61) and 25 years (7–70), respectively. Renal (74%) and gastrointestinal involvement (44%) were the most common, followed by skin(9%), pulmonary (9%), and cardiac involvement (9%). Primary antibody deficiencies(48%), combined immunodeficiencies(31%), hyperimmunoglobulin E syndrome(9%), congenital neutropenia (4%), autoinflammatory disorders (4%), and chronic mucocutaneous candidiasis (4%) were the IEI types associated with amyloidosis. Bronchiectasis (74%) and malignancy (17%) were observed in given ratio of patients. Treatment modalities for amyloidosis include colchicine (*n* = 12, 52%), steroids (*n* = 5, 22%) and tocilizumab (*n* = 2, 9%) without significant benefit. Thirteen patients (57%) died with a median age of 24 years (8–45), predominantly due to sepsis (52%). Familial Mediterranean fever (FMF) gene analysis was negative in all patients except for one, who had a heterozygous MEFV gene defect (M694V).

**Conclusion:**

AA amyloidosis in IEI is associated with severe morbidity and mortality. Early diagnosis and management of IEI are crucial to prevent amyloidosis development. However, colchicine appears ineffective once amyloidosis has occurred, highlighting the need for further research into early diagnostic biomarkers and novel treatment options.

## Introduction

Amyloidoses are a rare and heterogeneous group of disorders characterized by the deposition of insoluble amyloid fibrils in tissues, resulting in organ destruction. AA amyloidosis is a systemic amyloidosis syndrome caused by extracellular deposition of insoluble SAA protein fibrils due to the chronic inflammation. SAA is a protein secreted by hepatocytes in response to inflammatory stimuli such as TNF, IL-1, and IL-6. The accumulation of SAA can affect various organs such as the kidneys, heart, liver, gastrointestinal and respiratory system, bone marrow, skin, and muscles. Various diseases that cause chronic inflammation may trigger amyloidosis, the most common of which are chronic infections, autoimmune, inflammatory, rheumatological disorders, malignancies and inborn errors of metabolism [1, 2]. Inborn errors of immunity (IEI) are also associated with the development of amyloidosis. Chronic inflammation in IEI caused by infections and as well as immune dysregulatory nature of the disease, result in extracellular deposition of SAA. To date, as far as we know, only 40 cases of IEI with amyloidosis have been reported over 60 years, mostly in patients with primary antibody deficiency (70%), followed by combined immune deficiency (CID) (10%) and phagocytic system deficiency (10%) [3]. Treatment of the infection, immunomodulatory therapy and hematopoietic stem cell transplantation (HSCT) may reduce high mortality in IEI patients with amyloidosis.

The present study aimed to determine the clinical manifestations, immunological findings, medical approach and outcome of the patients diagnosed with IEI and AA amyloidosis.

## Methods

**Fig. 1:**
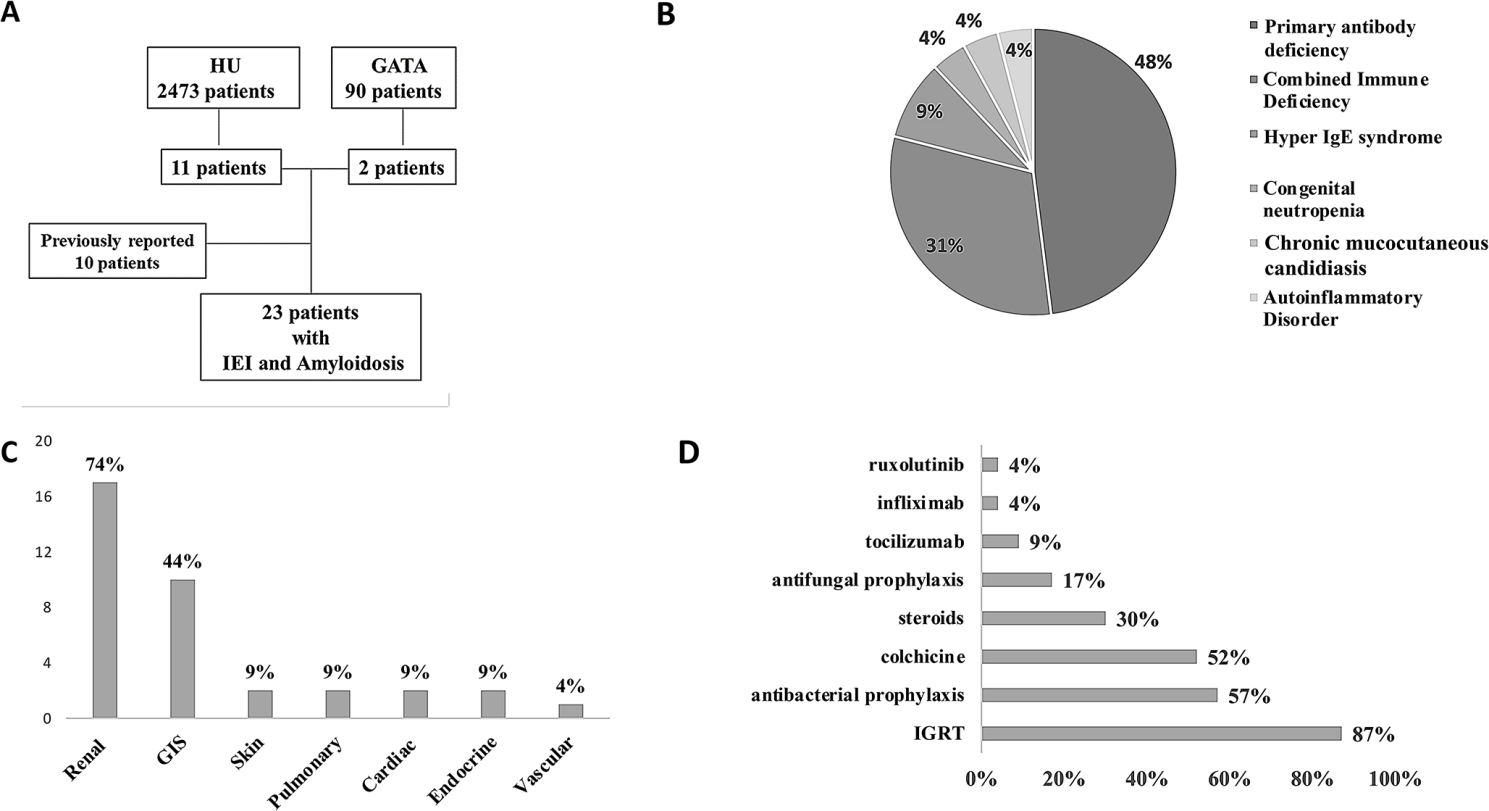
**A.** Scheme of the study design, **B. **IEI diagnoses of 23 patients with AA amyloidosis, **C.**Organ involvement in patients with amyloidosis, **D.**Treatments for AA amyloidosis and IEI. CMCC: chronic mucocutaneous syndrome, GATA: Gulhane Training and Research Hospital, GIS: gastrointestinal syndrome, HIES: Hyper IgE syndrome, HU: Hacettepe University, IGRT: immunoglobulin replacement therapy, PAD: Primery antibody deficiency

We reviewed thirteen patients diagnosed with IEI and AA amyloidosis between 2000 and 2022 at the Division of Pediatric Immunology, Hacettepe University Ihsan Dogramaci Children’s Hospital (n=11), and Gulhane Training and Research Hospital (n=2), retrospectively. During the same period, 2473 IEI patients have been followed up in the Division of Pediatric Immunology, Hacettepe University İhsan Doğramacı Children’s Hospital (Figure 1A).

The diagnosis of IEI was based on the European Society for Immunodeficiencies’s criteria (ESID.org, ESID Registry—Working Definitions for Clinical Diagnosis of PID). Demographic features, clinical characteristics, histological and laboratory manifestations, and therapeutic interventions of the patients were evaluated. Next-generation sequencing (NGS) for IEI was performed on seven (54%) out of 13 patients. HaloPlexTM probes which were designed to capture 356 IEI-related genes were used as previously described [4].

The AA amyloidosis diagnosis involving the pulmonary system, gastrointestinal system, skin, and kidney was confirmed through histopathological examination of Congo red staining identified amyloid deposits. To confirm the AA subtype, immunohistochemistry (IHC) for serum amyloid A (SAA) protein was performed on formalin-fixed, paraffin-embedded tissue sections. A diagnosis of a probable cardiac amyloidosis was based on the previously reported amyloidosis-associated echocardiographic findings (thickened left ventricular (LV) walls and a normal or reduced LV cavity size) supported by BNP levels [5].

Patients with renal involvement but not requiring dialysis (seven patients) were classified according to the kidney failure staging system for AA amyloidosis [6]. By including additional l0 patients reported from Türkiye, we analyzed the clinical characteristics and laboratory manifestations of a total of 23 cases in a compherensive way (Fig. 1A).

## Results

### Demographic Findings of the Patients

Thirteen patients with a male/female ratio of 10/3 were diagnosed with AA amyloidosis. The median follow-up period was three years (0.5–22) The prevalence of amyloidosis in IEI patients was 4.4/1000 (11/2473) in the Division of Pediatric Immunology, Hacettepe University İhsan Doğramacı Children’s Hospital. The median ages at symptom onset and IEI diagnosis were 6 (0.5–60) years and 21 years (2–61), respectively (Table 1). The median interval between symptom onset and IEI diagnosis was 9 years (1–37). Four patients had parental consanguinity, and three had a family history of IEI.

**Table 1:** Demographic and clinical features of the patients

	**P1**	**P2**	**P3**	**P4**	**P5**	**P6**	**P7**
**Gender**	Male	Female	Male	Female	Male	Male	Male
**Age onset of IEI symptoms (age)**	7	2.5	2	8	6	10	5
**Age at IEI diagnosis (age)**	15	9	34	36	15	21	27
**Age at AAA diagnosis (age)**	25	9	33	35	15	21	28
**IEI diagnoses**	HIES	ADA2 deficiency	STAT1 deficiency	CID	CID	STK4 deficiency	CVID
**IEI clinical features**							
Recurrent RTI	Yes	Yes	Yes	Yes	Yes	Yes	Yes
CMCC	-	-	Yes	-	-	-	-
Viral infections	-	-	-	HPV, VZV	-	HPV	-
Bronchiectasis	-	-	Yes	Yes	Yes	Yes	Yes
Malignancy	Papillary thyroid cancer	-	-	HL	-	NHL	-
**AAA manifestations**							
***First symptom of AAA***	Eczema	Facial edema	Elevated serum creatinine level	Diarrhea	Elevated serum creatinine level and dysphagia	Cough and dyspnea	Proteinuria
***AAA diagnosis before symptoms***	No	No	No	No	No	No	Yes
***Renal***	-	Nephrotic syndrome and renal failure	Nephrotic syndrome and renal failure	-	Nephrotic syndrome and renal failure	-	Non-nephrotic syndrome
***Gastrointestinal***	-	Colon and rectum	Colon	Colon	Esophagus and stomach	-	-
***Cardiac***	-	Probable (LVH)	-	-	Probable (LVH)	-	-
***Skin***	Yes	-	-	-	NA	-	-
***Pulmonary***						Yes	Yes
**FMF gen analysis**	M694V Heterozygous	Negative	Negative	Negative	Negative	Negative	Negative
**IEI treatments**	IGRT, antibacterial prophylaxis	IGRT, ruxolutinib and antibacterial prophylaxis	IGRT, antibacterial and antifungal prophylaxis	IGRT, antibacterial, antiviral and antifungal prophylaxis	IGRT, antibacterial prophylaxis	IGRT, antibacterial prophylaxis	IGRT, antibacterial and antifungal prophylaxis
**ESR at AAA diagnosis (mm/hr)**	11	53	75	45	32	120	47
**CRP (mg/dl)**	0.5	14.9	4.7	7.9	7.5	9.9	2.3
Proteinuria g/24hour	-	6.3	8.2	0.6	16	1.2	2.2
eGFR	> 90	34	50	60	25	51	60
**AAA treatments**	-	Colchicine, tocilizumab and steroids	Ruxolutinib, ACEI and hydroclorotiazid	-	Colchicine and tocilizumab		Colchicine and ARB
**Outcome**	Stable	Died of sepsis (2 months after AAA diagnosis)	Stable	Died of sepsis (1 year after AAA (diagnosis)	Died of intracranial hemorragia (2 years after AAA diagnosis)	Died of pneumonia (1 year after AAA diagnosis)	Died of pneumonia (1 year after AAA diagnosis)
	**P8**	**P9**	**P10**	**P11**	**P12**	**P13**	
**Gender**	Male	Male	Female	Male	Male	Male	
**Age onset of IEI symptoms**	12	60	1	7	3	6 months	
**Age at IEI diagnosis (years)**	40	61	4.5	44	12	2	
**Age at AAA diagnosis**	41	70	16	45	33	15	
**IEI diagnoses**	CVID	CMCC	Congenital neutropenia	CID	CID	HIES	
**IEI clinical features**							
Recurrent RTI	Yes	-	Yes	Yes	Yes	Yes	
CMCC	-	-	Yes	-	-	-	
Viral infections	-	-	-	CMV	-	-	
Bronchiectasis	-	-	-	Yes	Yes	No	
Malignancy	-	-	Papillary thyroid ca.		-		
Others	-	-	-	-	Autoimmune hypothyroidism	Asthma	
**AAA manifestations**							
***First symptom of AAA***	Weight loss	Pruritus, hyperpigmentation of the skin	Proteinuria	Proteinuria	Proteinuria, weigth loss and diarrhea	Proteinuria	
***AAA diagnosis before symptoms***	No	No	Yes	Yes	No	Yes	
***Renal***	-	-	Nephrotic syndrome	Nephrotic syndrome	Nephrotic syndrome	Nephrotic syndrome, hypertension	
***Gastrointestinal***	Duodenum	-	-	Stomach, duodenum, ileum, colon and rectum amyloidosis			
***Cardiac***	-	-	NA	-	-	NA	
***Skin***	-	Yes	-	-	-	-	
***Pulmonary***						-	
**FMF gen analysis**	NA	NA	NA	NA	NA	Negative	
**IEI treatments**	IGRT, antibacterial prophylaxis	Antifungal prophylaxis	GCSF, antibacterial prophylaxis	IGRT, antibacterial prophylaxis	IGRT, antibacterial prophylaxis	IGRT antibacterial prophylaxis	
**ESR at AAA diagnosis (mm/hr)**	6	2	NA	77	68	20	
**CRP (mg/dl)**	0.9	0.2	-	-	-	0.5	
Proteinuria gr/24 hours	0.2	0.08	6.2	2.5	7.2	6.1	
eGFR	> 90	> 90	32.5	> 90	> 90	> 90	
**AAA treatments**			Colchicine	Steroids	Colchicine, furosemide	Steroids, colchicine, ACE inh.	
**Outcome**	Stable	Stable	Lost to follow-up	Died of sepsis (2 weeks after AAA diagnosis)	Died of sepsis (1 months after AAA diagnosis)	Lost to follow-up	

AAA: AA amyloidosis, ACEI: angiotensin-converting enzyme inhibitor, ARB: angiotensin-receptor blocker CID: combined immune deficiency, CMCC: chronic mucocutaneous candidiasis, CRP: C-reactive protein, ESR: Erythrocyte sedimentation rate, CVID: common variable immune deficiency, eGFR: Estimated glomerular filtration rate FMF: Familial Mediterranean fever, HIES: Hyper IgE syndrome, HL: Hodgkin lymphoma, HPV: human papilloma virus, IEI: inborn errors of immunity, IGRT: immunoglobulin replacement therapy, LVH: left ventricular hypertrophy, NHL: non Hodgkin lymphoma, NA: not applicable, RTI: respiratory tract infection, VZV: varicella zoster virus

The median age at amyloidosis diagnosis was 28 years (9–70). The median time between onset of IEI symptoms and diagnosis of AA amyloidosis was 18 years (6.5–38). Concomitant diagnosis of IEI and AA amyloidosis was made in three patients (P2, P5 and P6).

### Inborn Errors of Immunity-related Manifestations of the Patients

Infections were the most common clinical feature of IEI, with twelve patients (92%) having a history of recurrent upper and lower respiratory system infections since childhood. Recurrent pneumonia led to bronchiectasis in seven patients (54%) and two patients (P5 and P7) underwent pulmonary lobectomy. Chronic mucocutaneous candidiasis (15%) and warts (15%) were observed during the follow-up.

Four (30%) of the patients had a history of malignancy. Two patients (15%) were previously diagnosed with lymphoma (P4, Hodgkin lymphoma; P6, Non-Hodgkin lymphoma), and two patients (P1 and P9) were diagnosed with papillary thyroid cancer. One patient (P12) developed Hashimoto thyroiditis during the follow-up.

### Inborn Errors of Immunity Diagnosis of the Patients

P2, P3, P4, P5, P6, P11, and P12 were diagnosed with CID, P1 and P13 were diagnosed with hyperimmunoglobulin E syndrome (HIES), P7 and P8 with common variable immunodeficiency (CVID), P9 with chronic mucocutaneous candidiasis (CMCC), and P10 with congenital neutropenia. P2 was diagnosed with ADA2 deficiency, P3 with STAT1 deficiency, and P6 with STK4 deficiency by NGS study. The diagnoses are shown in Fig. 1b.

### Amyloidosis-related Manifestations of the Patients

**Fig. 2:**
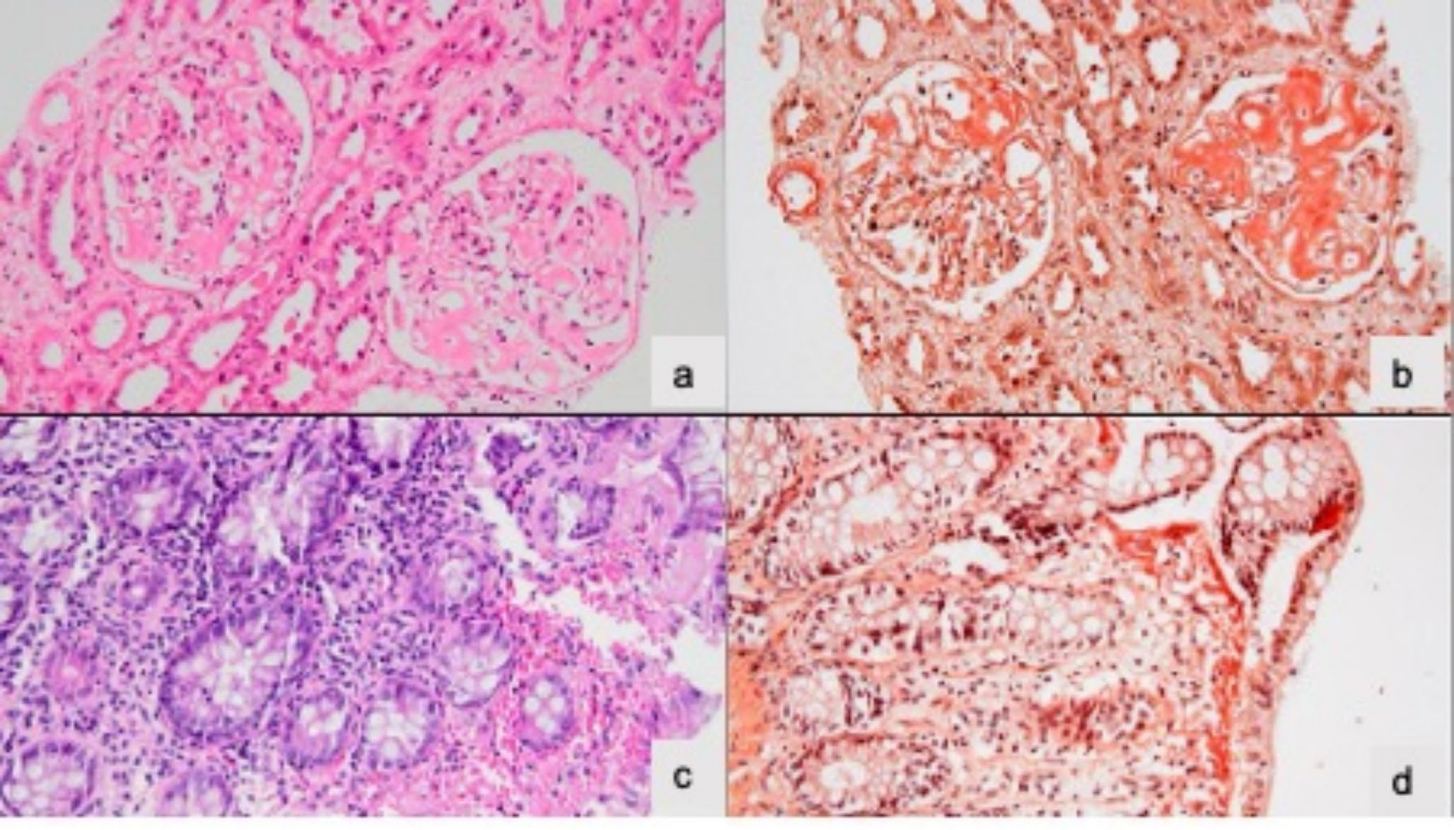
**A.** Kidney biopsy of P5; The mesangial area of the glomeruli is widened by amorphous homogenous eosinophilic material. **B.** Kidney biopsy of P5; Congo red stain demonstrating congophilic nature of the material consistent with amyloid, also highlighting presence of deposition along the capillary basement membrane and hilar arteriole **C.** Colon biopsy of P2; Homogenous amorphous eosinophilic material is present within the lamina propria underlying the surface epithelium **D.** Colon biopsy of P2; Congo red stain shows congophilia beneath the surface epithelium, consistent with amyloid deposition

Renal involvement was the most frequent presentation of AA amyloidosis (8/13, 62%, Table 1), with nephrotic syndrome in seven patients, renal failure in three patients, and non-nephrotic syndrome in one patient. Renal biopsies in all patients revealed AA amyloidosis type amyloid accumulation (see Figure 2A and B for kidney biopsy of P5). When patients with renal involvement but not requiring dialysis (7 patients) were classified according to the kidney failure staging system for AA amyloidosis, 2 patients (29%) were at high risk, 3 patients (42%) were at intermediate risk, and 2 patients (29%) were at low risk of renal failure.

Gastrointestinal system involvement was observed in six (46%) patients. P2, P4 and P11 suffered from chronic diarrhea. AA amyloid accumulation was present in the colon and rectum biopsies of P2 and colon biopsies of P3 and P4 (Fig. 2C&D). P5 developed progressive dysphagia, and gastrointestinal endoscopy revealed achalasia requiring myotomy. Esophageal and gastric biopsies were positive for AA amyloid accumulation. P8 presented with the weight loss, and AA amyloid accumulation was detected in the duodenal mucosa. P11 was admitted with diarrhea and weight loss; AA amyloidosis due to CMV infection was observed in stomach, duodenum, ileum, colon, and rectum.

Cutaneous involvement was present in two (15%) patients. P1 had eczema from childhood, and was followed up for HIES, while P9 had CMCC for several years. Skin biopsies performed on P1 and P9 due to hyperpigmentation demonstrated AA amyloid accumulation in both patients.

Among the eleven patients who underwent echocardiographic studies, two (P2 and P5) showed radiological features of probable cardiac amyloidosis. Pulmonary involvement was observed in two (15%) out of thirteen patients. P6 presented with cough and dyspnea, along with pulmonary nodular lesions in thoracic CT. Transbronchial biopsy via flexible fiberoptic bronchoscope showed AA amyloid accumulation.

### Laboratory Features of the Patients

The median CRP and ESR values at the time of amyloidosis diagnosis were 4.0 mg/dl (0.2–14.9) and 46 mm/hr (2-120), respectively. Four patients (31%) were diagnosed with AA amyloidosis at a pre-symptomatic stage following the detection of proteinuria during routine evaluations.

### Therapeutic Approach and Outcome

Following IEI diagnosis, we started all patients, except P9 and P10, immunoglobulin replacement therapy (IGRT). We gave antifungal prophylaxis to P9, and GCSF treatment with co-trimethoxasole prophylaxis to P10.

In patients who received therapy directed against AA amyloidosis, P7, P10 and P12 received colchicine; P5 received colchicine and tocilizumab; P2 received colchicine, tocilizumab and steroids; P11 received steroids; P13 received colchicine and steroids, and P3 received ruxolitinib (Fig. 1d). One patient (P5) underwent hemodialysis, and none were transplanted.

Seven out of eleven patients (54%) died during the follow-up period, with a median age of 22 years (9-36), while two patients (P10 and P13) lost to follow-up. Causes of death included sepsis (P2, P4, P11 and P12), pneumonia (P6, P7), and intracranial hemorrhage while on hemodialysis (P5).

### Overview of the Patients With Amyloidosis in Türkiye

Ten additional patients previously reported from Türkiye were included (Table 2), bringing the total number of cases to 23 for a comprehensive analysis of clinical characteristics and laboratory manifestations. Upon analysis of all amyloidosis patients with IEI, the median age of IEI diagnosis was 20 years (2–61), while the median age at the time of amyloidosis diagnosis was 25 years (7–70). 

**Table 2:** Demographic and clinical features of previously reported patients

	P14	P15	P16	P17	P18	P19	P20	P21	P22	P23
**Gender**	Male	Female	Female	Male	Female	Male	Male	Female	Female	Male
**Age onset of IEI symptoms**	14	9	Early childhood	10	Childhood	9	Infancy	Infancy	Infancy	15
**Age at IEI diagnosis**	28	29	12	16	24	20	12	6	3	37
**Age at AAA diagnosis**	28	29	20	24	24	29	12	10	7	37
**IEI diagnoses**	CVID	CVID	NLRP12 deficiency	CVID	CVID	CVID	HIGM Syndrome	HIGM Syndrome	HIGM Syndrome	CVID
**IEI clinical features**										
Recurrent RTI	Yes	Yes	Yes	Yes	Yes	Yes	Yes	Yes	Yes	Yes
CMCC	-	-	-	-	-	-	-	-	-	-
Viral infections	-	-	Yes	-	Frequent oral herpes	-	-	-	-	-
Bronchiectasis	-	Yes	Yes	Yes	-	-	Yes	-	-	Yes
Malignancy	-	-	-	-	-	-	-	-	-	
**Others**	Pulmonary TB, Recurrent gastroenteritis	Recurrent gastroenteritis	JIA, diarrhea, SM, diffuse LAP	Chronic diarrhea	Meningitis, PID, tuboovarian&vaginal abscess	Recurrent gastroenteritis, HSM	HSM, LAP	HSM, LAP	HSM, LAP	-
**AAA manifestations**										
***First symptom of AAA***	Weight loss and diarrhea	Edema	Diarrhea	Edema and dispnea	Edema and dyspepsia	Dyspepsia and diarrhea	Edema	Edema	Edema	Fever and tachycardia
***AAA diagnosis before symptoms***	No	No	No	No	No	No	No	No	No	No
**Renal**	Nephrotic syndrome	Nephrotic syndrome	Nephrotic syndrome	Nephrotic syndrome	Nephrotic syndrome	Renal failure, nephrotic syndrome	Renal failure, nephrotic syndrome	Renal failure	Nephrotic syndrome	-
**Gastrointestinal**	Esophagus, stomach, duodenum, colon, rectosigmoid	-	Intestinal amyloidosis	-	Duodenum	Duodenum	-	-	-	-
**Cardiac**	-	-	-	-	-	-	-	-	-	
**Skin**	-	-	-	-	-	-	-	-	-	
**Pulmonary**	-	-	-	-	-	-	-	-	-	
**Other**	-	-	-	Adrenal, vascular	-	-	-	-	-	Thyroid
**FMF gen analysis**	Negative	NA	NA	Negative	NA	Negative	Negative	Negative	Negative	Negative
**IEI treatments**	No	IGRT	IGRT	IGRT, antibacterial prophylactic	IGRT	IGRT	IGRT	IGRT	IGRT	IGRT
**AAA treatments**	Colchicine	ACEI and ARB, Colchicine	Prednisolone, infliximab	Colchicine, ARB, diuretic, methylprednisolone	ACEI	ACEI and ARB	Colchicine	Colchicine, peritoneal dialysis	Colchicine	-
**Outcome**	Persistent nephrotic syndrome	Stable	Died of sepsis (6 months after AAA diagnosis)	Dies of sepsis (2 months after AAA diagnosis)	NA	NA	Died of infection (2 months after AAA diagnosis	Died of multiorgan failure (10 months after AAA diagnosis)	Died of infection (1 year after AAA diagnosis)	Died of sepsis (3 months after AAA diagnosis)
**Authors**	Celik et al. [23]	Aydin et al. [24]	Borte et al. [25]	Arslan et al. [26]	Kadiroglu et al. [27]	Turkmen et al. [28]	Oner et al. [29]	Oner et al. [29]	Oner et al. [29]	Soysal et al. [30]

AAA: AA amyloidosis, ACEI: angiotensin-converting enzyme inhibitor, ARB: angiotensin-receptor blocker, CMCC: chronic mucocutaneous candidiasis, CRP: C-reactive protein, ESR: Erythrocyte Sedimentation Rate, CVID: common variable immune deficiency, eGFR: Estimated Glomerular Filtration Rate, FMF: Familial Mediterranean fever, HIGM: Hyper Immunoglobulin M, HSM: hepatosplenomegaly, IEI: inborn errors of immunity, IGRT: immunoglobulin replacement therapy, JIA: juvenile idiopathic arthritis, LAP: lymphadenopathy, NA: not applicable, PID: pelvic inflammatory disease, RTI: respiratory tract infection, SM: splenomegaly, TB: tuberculosis

Primary antibody deficiencies (PAD, eight CVID patients and three hyperimmunoglobulin M syndrome (HIGM) patients) were the most common IEI (48%), followed by CID (31%), HIES (9%), congenital neutropenia (4%), autoinflammatory disorder (4%), and CMCC (4%) (Fig. 1b). The diagnoses are present in Figure 1B.

Renal involvement was the most frequent presentation (74%), followed by gastrointestinal involvement (44%), skin (9%), pulmonary (9%) and cardiac involvement (9%)(Fig. 1C). Amyloidosis of the adrenal gland (P14) and thyroid (P20) were also present. Common initial clinical features were edema in renal amyloidosis, weight loss, diarrhea, and dyspepsia in gastrointestinal amyloidosis, and cough and dyspnea in pulmonary amyloidosis (Table 1).

 Recurrent respiratory diseases were observed in all but one patient (96%). Bronchiectasis was the most common complication (74%) associated with IEI and amyloidosis, followed by malignancy (17%). Twelve patients (52%) received colchicine for the treatment of amyloidosis, seven (30%) had antiproteinuric therapy, and 5 (22%) received steroids (Fig. 1D).

Thirteen patients (57%) died during the follow-up period with a median age of 24 years (8–45), and sepsis (52%) was the most common reason of death.

## Discussion

Chronic uncontrolled inflammation resulting from immune dysregulation secondary to infections, inflammatory/autoimmune diseases, and lymphoproliferative/tumoral disorders is known to cause amyloidosis. Chronic inflammatory conditions are also components of IEI, such as inflammatory bowel disease, interstitial lung disease, sarcoidosis, arthritis, vasculitis, nephritic and nephrotic syndrome, and dermatitis. Almost 25% of IEI patients have inflammation and autoimmunity [7]. The mechanisms triggering inflammation in IEI include exacerbated production of type I interferon and IL-1, increased B and/or T cell activation, defects in regulatory T and B cells, and defects in the clearance of immune complexes, and apoptotic cell bodies. The major challenge is ongoing inflammation resulting from these mechanisms since misdiagnosis and delay in the diagnosis and treatment are common in cases of IEI.

In addition to CID and CVID [7], other IEI classes predispose individuals to inflammation. In this study, PAD (CVID in eight patients, and hyperimmunoglobulin M syndrome in three patients) were observed in majority of patients, followed by CID HIES, and innate immune system deficiency.

AA amyloidosis, the major complication of periodic fever syndromes, has also been reported in IEI. Patients may be misdiagnosed in regions where periodic fever syndromes are common, such as Türkiye, where the prevalence of FMF is about 1/1000 [8]. The prevalence of amyloidosis in FMF patients is approximately 8% [9], whereas it is 4.4 per 1000 in IEI patients.

Uncontrolled immune dysregulation due to the IEI mentioned at the beginning of the discussion may occur earlier in life, resulting in early-onset amyloidosis presenting at a younger age in contrast to periodic fever syndromes. In this study, the median age of amyloidosis diagnosis was 25 years (7–70). Blank et al. reported that the median ages at time of AA amyloidosis in FMF, monogenic autoinflammatory diseases and rheumatic diseases were 37, 34 and 55 years, respectively [10].

There is no specific treatment for AA amyloidosis. Therefore, the treatment of AA amyloidosis in IEI consists of two steps: controlling inflammation to prevent the development of amyloidosis, and treating amyloidosis once it has occurred. The second could not be performed effectively in this study despite many biologics under use. A 9-year diagnostic delay in IEI, leading to uncontrolled inflammation and infection, appears to be the cause of amyloidosis. Amyloidosis of the skin and gastrointestinal tract developed due to CMCC in P9 and CMV gastroenteritis in P11 while the inherent immune dysregulatory nature of the diseases seems to be the reason of amyloidosis in P1 and P3. Standard treatments, such as IGRT and antimicrobial prophylaxis, may not adequately control persistent inflammation in IEI [11]. That is why prompt initiation of immunosuppressive treatment following recognition of initial signs of chronic inflammation, such as non-infectious bronchiectasis, inflammatory lung disease (ILD), granuloma, inflammatory bowel disease (IBD) and enteropathy, is necessary to delay or slow the natural course of AA amyloidosis. Targeted therapies based on the underlying mechanism of disease control hyperinflammation and immune dysregulation, such as abatacept in CTLA-4 and LRBA deficiency, tocilizumab in STAT3-GOF, and sirolimus in activated phosphoinositide 3-kinase d syndrome (APDS) [12]. HSCT may also be considered early in the course of the disease to control inflammation in indicated IEI [13].

In this study, only three patients received treatment specific to AA amyloidosis other than colchicine or steroids, with two receiving tocilizumab and one receiving ruxolutinib. Various drugs are effective in AA amyloidosis treatment depending on the underlying pathogenic mechanisms. In FMF, colchicine inhibits the synthesis of acute phase proteins [14, 15]. However, when renal failure and severe nephrotic syndrome develop, colchicine does not seem to be effective. Similarly, while colchicine does not reduce amyloid deposition in amyloidosis secondary to inflammatory bowel disease [16]. However, tocilizumab and 5-Mercaptopurine have shown promising results in these cases [16, 17]. Furthermore, in other periodic fever syndromes, such as TRAPS, CAPS and HIDS, IL-1 receptor antagonist anakinra and anti-TNF agents have proven effective in controlling symptoms, whereas colchicine is ineffective in preventing and treating attacks [18]. Tocilizumab has been shown to be effective for AA amyloidosis secondary to rheumatic diseases including rheumatoid arthritis and juvenile idiopathic arthritis [19]. In summary, once amyloidosis has developed, colchicine mostly seems to be ineffective, and treatment with new therapotic options should be considered to control inflammation and potentially reverse amyloid deposition without delay.

In this study 57% of patients died. Similarly, a previous study, reported a 50% mortality rate in IEI patients with AA amyloidosis [3], indicating that the presence of AA amyloidosis in IEI predicts poor prognosis. Nephrotic syndrome, end-stage renal disease, and elevated SAA concentrations, primarily linked to the severity of inflammation, and increase mortality in AA amyloidosis [1, 20, 21]. Early diagnosis of AA amyloidosis prior to the onset of clinical manifestations is crucial for preventing disease progression and reducing mortality, as only 31% of patients in this study were diagnosed at a pre-symptomatic stage, with the majority already exhibiting irreversible organ damage by the time clinical symptoms developed. Staging AA amyloidosis can help identify high-risk patients who would benefit from closer monitoring and intensive treatment to control inflammation. Furthermore, regular assessment of eGFR and 24-hour proteinuria seem to be key prognostic factors for renal failure evaluation, while BNP measurements and low eGFR may provide critical insights into overall survival [6].

In this study, SAA measurement was not available, but median CRP and ESR values at the time of amyloidosis diagnosis were 4.0 mg/dl and 46 mm/hr, respectively. Although CRP is commonly used as an alternative when SAA measurement is not feasible [22], and ESR is an indirect marker of inflammation that rises gradually and takes weeks to normalize, ESR seems to be more effective than CRP in detecting chronic inflammation in this study. Furthermore, elevated SAA levels correlate with the progression of AA amyloidosis [1, 22]. Therefore, close monitoring of SAA levels and normalization into the reference range may help control inflammation, and assess treatment efficacy. However, SAA levels may not accurately represent the degree of organ dysfunction, emphasizing the need for more specific diagnostic biomarkers.

As AA amyloidosis is rare in IEI, routine ESR or SAA monitoring for all patients may not be cost-effective. However, ESR or SAA monitoring should be tested in the routine follow-up at 3-month intervals in patients having IEI and chronic, uncontrolled, and persistent inflammation. Persistently elevated acute phase reactants, reduced eGFR and new-onset proteinuria should be recognized as potential warning signs of AA amyloidosis, requiring further evaluation. Manifestations involving the two most frequently affected systems in amyloidosis—renal involvement, presenting as edema, and gastrointestinal involvement, characterized by dyspepsia, diarrhea, and weight loss should be regarded as critical indicators requiring further evaluation. Moreover, we suggest that amyloidosis should be regarded as a severe complication of the underlying IEI, rather than the coexistence of a secondary periodic fever syndrome, to avoid delays in managing inflammation and initiating appropriate treatment.

## Conclusion

In summary, although AA amyloidosis appears to be rare in IEI, it is associated with severe morbidity and mortality. Prevention of AA amyloidosis in IEI depends on early diagnosis of IEI and treatment of underlying inflammatory causes such as infections. Persistently elevated acute phase reactants, new-onset proteinuria, and elevated creatinine levels are critical for early diagnosis of amyloidosis. Further research is needed to identify reliable diagnostic biomarkers and develop clinically relevant staging system for AA amyloidosis. Once amyloid deposition has occurred, colchicine seems to be ineffective. Therapeutic approaches for preventing polymerisation of amyloid fibrils and their deposition are necessary, and will be promising in amyloidosis treatment in the future.

## Data Availability

Data is provided within the manuscript.

## Abbreviations

AA: Amyloid A
APDS: Activated phosphoinositide 3-kinase delta syndrome
CID: Combined immunodeficiencies
CMCC: Chronic mucocutaneous candidiasis
CRP: C-reactive protein
CVID: Common variable immunodeficiency
ESR: Erythrocyte sedimentation rate
ESID: European Society for Immunodeficiencies
HIGM: Hyperimmunoglobulin M syndrome
HIES: Hyperimmunoglobulin E Syndrome
HSCT: Hematopoietic stem cell transplantation
IEI: Inborn Errors of Immunity
IL-1: Interleukin 1
IL-6: Interleukin 6
NGS: Next-generation sequencing
PAD: Primary antibody deficiencies
SAA: Serum Amyloid A
TNF: Tumor Necrosis Factor
